# Supplementary material for: An Interval Two-Stage Stochastic Programming Model for Flood Resources Allocation under Ecological Benefits as a Constraint Combined with Ecological Compensation Concept
Source: Int J Environ Res Public Health. 2019 Mar 21;16(6):1033. doi: 10.3390/ijerph16061033 (PMC6466455; doi:10.3390/ijerph16061033)
Supplement: Supplementary file 1 [file ijerph-16-01033-s001.pdf]

Supplementary Materials

An Interval Two-stage Stochastic Programming Model for Flood Resources Allocation under Ecological Benefits as a Constraint Combined with Ecological Compensation Concept

A. Overview of the Momoge National Nature Reserve (MNNR)

The West Water Supply Project of Jilin Province is an ecological water transfer project, which is both a water conservation project and an ecological project. According to "Planning report on comprehensive utilization of rivers and lakes for rainwater and flood resources in western Jilin Province", water diversion is given priority to ensure the water needs of the MNNR. Therefore, 15 lakes in the MNNR are selected for this study. The three water intakes for the Momoge Nature Reserve are the third branch channel of the Baishatan irrigation area (TBC), the Shijianfang intake gate (SIG) and the Haernao pumping station (HPS).

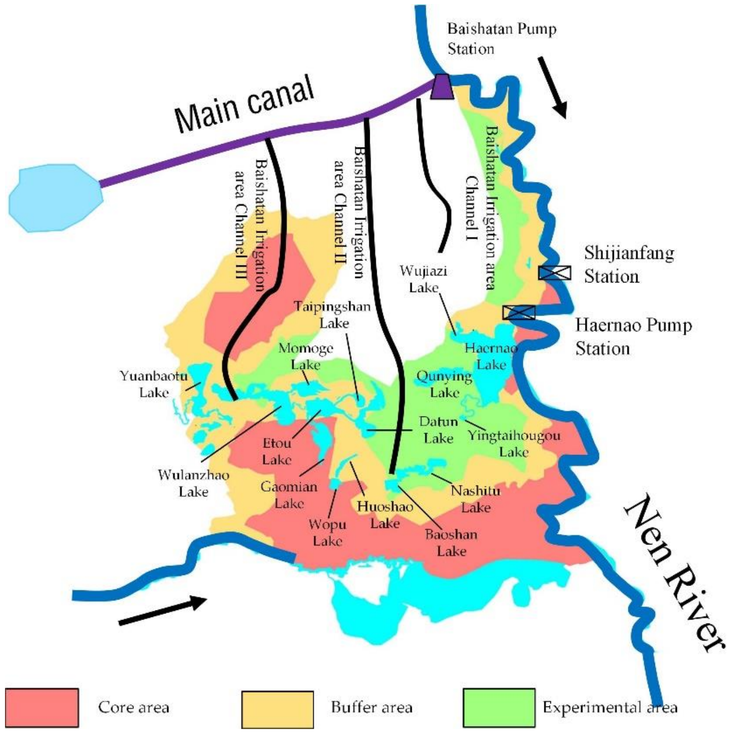

Figure S1. Overview of the MNNR.

B. The evaluation method of the indicator layer

Table S1. Evaluation method of wetland ecological service function evaluation index system.

Indicator Layer	Evaluation Method
Food production value	Market valuation evaluation
Raw material production value	Market valuation evaluation
Carbon sequestration value	Avoidable cost evaluation
Oxygen release value	Market valuation evaluation
Flood storage value	Alternative cost evaluation

Microclimate regulation value	Reference
Plant adsorption value	Reference
Biodiversity value	Reference
Scientific research value	Reference
Tourism value	Contingent value method
Landscape value	Reference

The value of the indicator layer is calculated as follows.

1. The food production value of fish and crabs can be calculated by market value evaluation:

$$V_F = \sum_{i=1}^{n} A_i \times Y_i \times P_i \quad (S1)$$

where V_F is food production value, Yuan; A_i is farming area, m^2; Y_i is the yield of food i, kg/m^2; P_i is the price of food i, Yuan/kg. Fish and crab production is calculated at 0.12 kg/m^2. The unit price of fish is 20 Yuan/kg, and the unit price of crab is 30 Yuan/kg.

2. Raw material production

The production value of raw materials of reed can be calculated by market value evaluation [1]:

$$V_M = A \times C \times P_m \quad (S2)$$

where, V_M is the production value of reed, Yuan; A is the area of reed m^2; C is the yield per unit area of reed, kg/m^2; P_m is the price of reed. The reed yield and unit price are 0.45 kg/m^2 and 0.4 Yuan/kg, respectively.

3. Carbon sequestration value

Plants in wetlands can absorb carbon dioxide through photosynthesis. The wetland (excluding waters) in the study area includes reed wetlands, marsh wetlands, etc. The biomass of reed wetlands is 0.45 kg/m^2, and the biomass of marsh wetlands is 0.07 kg/m^2, and the biomass of three schemes is calculated. The calculation of plant carbon sequestration in wetland ecosystems can reference the photosynthesis equation that per 1 g of dry matter synthesized can absorb 1.63 g of carbon dioxide, or 0.44 g of carbon.
The carbon sequestration value of wetlands is determined by avoidable cost evaluation [2].

$$V_C = W_C \times P \quad (S3)$$

where V_C is the carbon sequestration benefit of the wetland, Yuan; W_C is carbon sequestration in plants, t; P is the unit price of carbon sequestration, Yuan/t. The price of carbon is calculated at 277.7 Yuan/t.

4. Oxygen release

Wetland plants can regulate the atmosphere through oxygen produced by photosynthesis. Referring to the photosynthesis equation, 1.2 g of oxygen can be produced for each 1 g of dry matter. The atmospheric regulation value of wetlands can be determined by market value method:

$$V_O = 1.2 \times W \times P_O \quad (S4)$$

where V_O is the oxygen release benefits, Yuan; W is oxygen release mass, t; P_O is the price of oxygen per unit mass, Yuan/t. The unit oxygen price is calculated based on the industrial oxygen price of 1000 Yuan/t.

5. Regulating floods

The value of flood storage is determined by the alternative cost evaluation [3].

$$V_r = (W_s + W_r) \times P_r \quad \text{(S5)}$$

where V_r is flood storage value, Yuan; W_S is the wetland flood storage capacity, m^3/m^2; W_r is the lake flood storage capacity, m^3/m^2; P_r is the cost of the reservoir, Yuan/m^3. The wetlands can be stored with flood of 0.81 m^3/m^2. The lakes can be stored with flood of 0.67 Yuan/m^3.

6. Microclimate regulation

Wetlands can adjust the humidity and temperature of the area. According to the research results of Xie et al. [4] and the influence of factors such as inflation in recent years, and referring to the changes in the output value and food consumption level of China's crops from 2003 to 2012. Based on this calculation, the climate regulation value of lakes is 0.07 Yuan/m^2, and the climate regulation value of the wetlands is 3.64 Yuan/m^2.

7. Plant adsorption

Wetlands are the most purified ecosystems, and plants in wetland ecosystems can absorb pollutants from water bodies. The project will restore a large area of wetland, and the restored wetland will have an important value for purification. According to the research results of Xie et al. [4], and referring to the changes in the output value of China's agricultural products and the consumer price of food in 2003–2012, it is estimated that the value of wetland plant adsorption is 2.80 Yuan/m^2.

8. Biodiversity

The biodiversity value of wetlands is calculated using the results based on reference.

$$V_B = A \times P_B \quad \text{(S6)}$$

where V_B is the value of biodiversity, Yuan; A is the area of wetlands, m^2; P_B is the biodiversity value per unit area, Yuan/m^2. According to the research of Costanza et al. [5], the global biodiversity value per unit area of wetland is 0.20/m^2.

9. Scientific research value

Lakes and wetlands in the west of Jilin Province are effective places for research biology, ecology, and environmental science. This paper uses the results reference method to determine the scientific and cultural value of wetlands.

$$V_S = A \times P_S \quad \text{(S7)}$$

where V_S is scientific research, Yuan; A is area of wetlands, m^2; P_S is scientific research value per unit area, Yuan/m^2. The average scientific research value per unit area of wetlands in China is 0.04 Yuan/m^2 [6].

10. Tourism development value

Because of its rare species and unique environment, community and landscape, wetland ecosystems are often used as tourism and leisure places, and people can benefit from tourism opening. The open value of tourism can be determined by the contingent value method [3].

$$V_T = A \times P_T \quad \text{(S8)}$$

where V_T is tourism value, Yuan; A is the wetland area, m^2; P_T is tourism value per unit area, Yuan/m^2. The average tourism value of wetlands per unit area is calculated as 0.55 Yuan/m^2.

11. Landscape value

The aesthetic value of the wetland landscape can be determined using the results reference method.

$$V_L = A \times P_L \quad \text{(S9)}$$

where V_L is wetland landscape value, Yuan; A is the wetland area, m^2; P_L is landscape value per unit area, Yuan/m^2. The average landscape value per unit area of wetland is calculated as 0.21 Yuan/m^2 [4].

According to Equation (S1)–(S9), the unit ecological function service value (B) can be obtained; according Equation (1) in the manuscript, the ecological benefit of the "*Planning Report*" is:

$$f = \sum_{i=1}^{15}\sum_{j=1}^{4} A_{ij} \cdot Y_{ij} \cdot B_j - \sum_{m=1}^{2}\sum_{n=1}^{3} T_{mn} \cdot W_{mn} \quad \text{(S10)}$$

where Y and T have the same meaning as the Equation (1) in the manuscript, A and W are the planned area and water quantity.

Reference

1. Zhang, T., Che, L., Pu, B., et al. Evaluation of Lalu Wetland ecosystem services in Lhasa, Tibet [J]. Acta Ecologica Sinica, **2005**, 12, 3176-3180.
2. Pang, B., Cui, L., Ma, M., et al. Evaluation of Ecosystem Services Valuation of Alpine Wetlands in Zoigê Plateau [J]. Wetland Science **2014**, 12, 273-278.
3. Li, W., Cui, L., Pang, B., et al. Thinking of solving double counting in wetland ecosystem service Valuation [J]. Ecology and Environmental Science, **2014**, 23, 1716-1724.
4. Xie, G., Zhen, L., Lu, Q., et al. Expert Knowledge Based Valuation Method of Ecosystem Services in China [J]. Journal of Natural Resources, **2008**, 5, 911-919.
5. Costanza, R., Perrings, C., Cleveland C.J. The development of ecological economics [J]. Journal of Business Administration & Policy Analysis, **1997**. Available at https://www.researchgate.net/publication/241019788_THE_DEVELOPMENT_OF_AN_ECOLOGICAL_ECONOMICS.
6. Pang, B. **2014**. Research on Deduplication Calculation of Wetland Ecosystem Service Value Evaluation. Ph. D. dissertation, Chinese Academy of Forestry, Beijing, China.

C. The ecological benefit per unit area

The algorithm for the ecological benefit per unit area can be found in Question 1. The calculation results are shown in Table S2.

Table S2. Ecological benefit per unit area (Yuan/m^2).

	Fish Pond	Crab Pond	Reed	Marsh
Food production (Fish)	2.40	-	-	-
Food production (Crab)	-	3.60	-	-
Raw material production (Reed)	-	-	0.18	-
Carbon sequestration	-	-	0.05	0.05
Oxygen release	-	-	0.54	0.54
Flood storage	0.54	0.54	0.78	0.78
Microclimate regulation	0.07	0.07	3.64	3.64
Plant adsorption	-	-	2.80	2.80
Biodiversity	0.20	0.20	0.20	0.20
Scientific research	0.04	0.04	0.04	0.04
Tourism	0.55	0.55	0.55	0.55
Landscape	0.21	0.21	0.21	0.21

For different water intakes, the ecological functional areas are different; for different ecological functional areas, the types of ecological services are different. We did not use weights to estimate the water shortage penalty coefficient, perhaps this is a better approach. However, according to the eco-functional area category corresponding to each water intake, we use the maximum and minimum values of the corresponding service value of the ecological functional area to indicate the water shortage penalty coefficient of the ecological functional area. Therefore, the water shortage penalty coefficient of TBC, SIG and HPS are all [0.04, 3.64] (Yuan/m^3).
